# Association Between Bipolar Disorder or Schizophrenia and Oral Anticoagulation Use in Danish Adults With Incident or Prevalent Atrial Fibrillation

**DOI:** 10.1001/jamanetworkopen.2021.10096

**Published:** 2021-05-17

**Authors:** Morten Fenger-Grøn, Claus Høstrup Vestergaard, Anette Riisgaard Ribe, Søren Paaske Johnsen, Lars Frost, Annelli Sandbæk, Dimitry S. Davydow

**Affiliations:** 1 Research Unit for General Practice, Aarhus Denmark; 2 Department of Public Health, Aarhus University, Aarhus, Denmark; 3 Danish Center for Clinical Health Services Research, Department of Clinical Medicine, Aalborg University, Aalborg, Denmark; 4 Department of Clinical Medicine, Aarhus University, Silkeborg Regional Hospital, Silkeborg, Denmark; 5 Steno Diabetes Center, Aarhus University Hospital, Aarhus, Denmark; 6 Comprehensive Life Resources, Tacoma, Washington

## Abstract

**Importance:**

Individuals with bipolar disorder or schizophrenia have a higher risk of adverse outcomes from cardiovascular diseases. Oral anticoagulation therapy (OAT) for patients with atrial fibrillation (AF) is needed for stroke prevention, but whether patients with bipolar disorder or schizophrenia face disparities in receiving this therapy is unknown.

**Objective:**

To assess whether bipolar disorder or schizophrenia is associated with a lower rate of OAT initiation in patients with incident AF and lower prevalence of OAT in those with prevalent AF.

**Design, Setting, and Participants:**

A nationwide cohort study of Danish patients with AF was conducted from January 1, 2005, to December 31, 2016, and data were analyzed from January 1 to June 15, 2020. Data from national registries included information on all redeemed prescriptions and all hospital contacts of all patients with incident or prevalent AF (age, 18-100 years) and increased risk status, defined by a CHA_2_DS_2_-VASc (congestive heart failure, hypertension, age ≥75 years, diabetes, stroke or transient ischemic attack, vascular disease, age 65-74 years, sex category) risk score greater than or equal to 2.

**Exposures:**

Hospital diagnosis of bipolar disorder or schizophrenia.

**Main Outcomes and Measures:**

Adjusted proportion differences for OAT initiation and OAT prevalence, comparing individuals with and without bipolar disorder or schizophrenia.

**Results:**

Patients included with incident AF (n = 147 810) had a mean (SD) age of 76.9 (10.1) years, 78 577 (53.2%) were women, 1208 (0.8%) had bipolar disorder, and 572 (0.4%) had schizophrenia. Accounting for age, sex, and calendar time, bipolar disorder and schizophrenia were associated with significantly lower frequency of OAT initiation within 90 days after incident AF (bipolar disorder: −12.7%; 95% CI, −15.3% to −10.0%; schizophrenia: −24.5%; 95% CI, −28.3% to −20.7%) and lower OAT prevalence in patients with prevalent AF (bipolar disorder: −11.6%; 95% CI, −13.9% to −9.3% schizophrenia: −21.6%; 95% CI, −24.8% to −18.4%). Adjusting for socioeconomic factors and other comorbid conditions attenuated these associations, particularly for patients with bipolar disorder. However, schizophrenia continued to be associated with a with a lower rate of OAT initiation (−15.5%, 95% CI, −19.3% to −11.7%) and a −12.8% (95% CI, −15.9% to −9.7%) lower OAT prevalence. These associations were also present after the introduction of non–vitamin K antagonists (adjusted proportion difference in 2013-2016: −12.4%; 95% CI, −18.7% to −6.1% for initiation and −10.1%; 95% CI, −13.8% to −6.4% for prevalence).

**Conclusions and Relevance:**

In this study, patients with bipolar disorder or schizophrenia were less likely to receive OAT in the setting of AF. For patients with bipolar disorder, this deficit was largely associated with socioeconomic factors and comorbidities, especially toward the end of the study period. For patients with schizophrenia, disparities in this stroke prevention therapy persistently exceeded what could be explained by other patient characteristics.

## Key Points

**Question:**

Is bipolar disorder or schizophrenia associated with lower use of anticoagulation therapy, which is a guideline recommendation for stroke prevention in patients with atrial fibrillation?

**Findings:**

In this nationwide cohort study of more than 150 000 Danish patients with atrial fibrillation, comorbid bipolar disorder and schizophrenia were associated with a lower likelihood of receiving anticoagulation. Particularly for patients with schizophrenia, this treatment deficit persistently exceeded that explained by socioeconomic characteristics or additional comorbidity, although improvement was seen after new oral anticoagulants were introduced.

**Meaning:**

Patients with psychiatric comorbidity may face disparity in stroke prevention after a diagnosis of atrial fibrillation, but results of this study suggest that these patients may benefit from increasing access to newer oral anticoagulants.

## Teaser

This cohort study examines the use of oral anticoagulation therapy in patients with bipolar disorder or schizophrenia who develop atrial fibrillation.

## Introduction

An extensive body of literature has identified that individuals with bipolar disorder or schizophrenia have a lower life expectancy compared with the general population.^1^ Most of this excess mortality is attributable to increased risk of developing, and subsequently dying of, chronic medical conditions, such as cardiovascular diseases.^1,2,3,4^

Atrial fibrillation (AF) is one of the most common cardiovascular illnesses,^5,6^ affecting approximately 1 in 3 persons in the industrialized world.^7^ Atrial fibrillation is associated with lower quality of life,^8^ heart failure,^9^ dementia,^10,11^ and a 5-fold increased risk of ischemic stroke,^12^ ultimately implying substantial mortality. Currently, stroke is the second leading cause of death and the third leading cause of disability worldwide.^13^ Atrial fibrillation–related stroke can be prevented by oral anticoagulation therapy (OAT),^14,15^ which is recommended for all individuals at increased risk of thromboembolic events, defined by a CHA_2_DS_2_-VASc (congestive heart failure, hypertension, age ≥75 years, diabetes, stroke or transient ischemic attack, vascular disease, age 65-74 years, sex category) risk score greater than or equal to 2.^16,17^

However, optimal adherence to OAT can be resource intensive, and even in populations with relatively good health care access, many patients with AF do not receive OAT.^18^ Oral anticoagulation therapy could be particularly challenging for patients with bipolar disorder or schizophrenia, because they have difficulties adhering to treatments for chronic conditions^19,20^ and poorer self-management.^21,22^ Yet, patients with severe mental illness, including bipolar disorder and schizophrenia, have an increased risk of stroke,^23,24^ making OAT particularly important for those with comorbid AF. However, data are sparse on OAT use among individuals with bipolar disorder or schizophrenia and comorbid AF. Prior studies have noted that patients with comorbid psychiatric illnesses and AF may be less likely to start OAT,^25,26,27^ but it remains unknown whether this observation reflects disparity beyond what can be related to sociodemographic factors or comorbidity. It is also unknown whether a possible treatment deficit may increase by time since diagnosis, and whether implementation of non–vitamin K antagonist oral anticoagulant (NOAC) therapy has affected this population. Non–vitamin K antagonist oral anticoagulant therapy may facilitate adherence owing to higher safety and fewer monitoring requirements compared with vitamin K antagonists—aspects that could be valuable for patients with psychiatric comorbidity. However, a previous overview suggested that treatment advances may widen health disparities.^28^

The present study aimed to explore whether bipolar disorder or schizophrenia is associated with OAT initiation within 90 days after hospital discharge with an incident AF diagnosis or with OAT prevalence in individuals with prevalent AF when adjusting for socioeconomic characteristics and comorbidity. We hypothesized that having bipolar disorder or schizophrenia would be associated with a lower likelihood of OAT initiation among individuals with incident AF and lower OAT prevalence among those with prevalent AF.

## Methods

### Design, Setting, and Participants

We used nationwide Danish registries (eMethods in the Supplement provide data sources) to identify all patients with incident or prevalent AF from January 1, 2005, to December 31, 2016, and assess use of OAT from redeemed prescriptions. The cohort was restricted to patients aged 18 to 100 years, had 5 or more years of uninterrupted residence in Denmark at the date of AF diagnosis, and were at increased risk of thromboembolic events. This study was approved by the Danish Data Protection Agency, the Danish Health Data Authority, and Statistics Denmark. According to Danish law, entirely register-based studies require no further ethical approval or informed consent. This study followed the Strengthening the Reporting of Observational Studies in Epidemiology (STROBE) reporting guideline.

Atrial fibrillation was defined as inpatient or outpatient contacts registered with codes 427.93 or 427.94 according to the *International Classification of Diseases, 8th Revision* or code I48 according to the *International Statistical Classification of Diseases, 10th Revision* (*ICD-10*). We did not include AF diagnoses made in emergency departments owing to insufficient validity.^29^

Increased risk status was defined as a CHA_2_DS_2_-VASc score greater than or equal to 2^16^ and evaluated at relevant index dates using registry-based data (eTable 1 in the Supplement). To examine OAT initiation, we included patients starting the date they left the hospital with an incident AF diagnosis, ie, their first AF diagnosis in the register history, and defined this as the study index date. Oral anticoagulation therapy initiation was assessed 0 to 180 days after this date; multivariable-adjusted comparisons focused on the uptake within 90 days. To accommodate 90 days of follow-up, the inclusion period ended September 30, 2016.

To ascertain the prevalence of OAT, we included patients with prevalent AF and assessed their treatment status annually, thus allowing multiple entries for patients with prevalent AF for 2 years or more and including patients with AF diagnosed before the study period. For these analyses, index dates were the dates of each assessment.

Exclusion (or censoring) criteria included mitral stenosis or mechanical prosthetic heart valves, left atrial appendage closure, coagulation defect, or heparin therapy; precise coding definitions for these criteria are included in eTable 2 in the Supplement. Otherwise, patients were followed up until death, emigration, or the end of the study, whichever came first.

Exposures of interest were bipolar disorder (*ICD-10*: F30-F31 or Anatomical Therapeutic Classification [ATC]: N05AN) and schizophrenia (or schizoaffective disorder) (*ICD-10*: F20 and F25) diagnosed in a general or psychiatric hospital.

### Outcomes

Outcomes of interest were initiation and prevalence of OAT, ie, vitamin K antagonists (ATC: B01AA) or NOAC (ATC: B01AE07, B01AX06/B01AF01, B01AF02, or B01AF03). Patients registered with both vitamin K antagonist and NOAC therapies were classified according to their latest redemption. For the assessment of OAT prevalence, we assumed a treatment duration after each prescription redemption of either 1 day per pill, 1 day per 2 pills (ATC: B01AE07 [dabigatran] and B01AF02 [apixaban]), or 2 days per pill (ATC: B01AA04 [phenprocoumon]) plus a 25% grace period (allowing leeway for prescription refilling) and duration of any intermediate hospitalizations. In sensitivity analyses, the dosage assumption was changed to 1 pill per day for all types of OAT, and the grace period was changed from 25% to 0%, 50%, and 100%.

### Covariates

Using previously developed approaches^30,31^ (eTable 1 and eTable 3 in the Supplement), we assessed baseline information on all covariates listed in Table 1 (ie, CHA_2_DS_2_-VASc conditions, sociodemographic factors, psychiatric comorbidities, and conditions or medical treatments of particular relevance to the HAS-BLED [hypertension, abnormal kidney or liver function, stroke, bleeding history or disposition, labile international normalized ratio, age ≥65 years, drug or alcohol use predisposing to bleeding] risk score).^16,32^

**Table 1.** Characteristics of All Patients With Incident and Prevalent AF With Increased Risk Status

Characteristic	No. (%)					
	Treatment initiation (patients)			Treatment prevalence (entry years)		
	Bipolar disorder	Schizophrenia	Full cohort	Bipolar disorder	Schizophrenia	Full cohort
Total, No.	1208	572	147 810	7954^a^	3259^b^	1 002 721^c^
Age, mean (SD), y	74.55 (10.27)	69.26 (12.37)	76.9 (10.1)	73.99 (10.37)	67.46 (12.82)	75.85 (10.26)
Age group, y						
<60	88 (7.3)	114 (19.9)	7728 (5.2)	1894 (23.8)	1336 (41.0)	187 935 (18.7)
60-69	303 (25.1)	167 (29.2)	27 677 (18.7)	2600 (32.7)	954 (29.3)	301 710 (30.1)
70-79	440 (36.4)	181 (31.6)	51 858 (35.1)	2299 (28.9)	724 (22.2)	324 838 (32.4)
80-89	311 (25.7)	92 (16.1)	47 806 (32.3)	1076 (13.5)	228 (7.0)	168 130 (16.8)
≥90	66 (5.5)	18 (3.1)	12 741 (8.6)	85 (1.1)	17 (0.5)	20 108 (2.0)
Sex^d^						
Female	785 (65.0)	355 (62.1)	78 577 (53.2)	4729 (59.5)	1832 (56.2)	488 644 (48.7)
Male	423 (35.0)	217 (37.9)	69 233 (46.8)	3225 (40.5)	1427 (43.8)	514 077 (51.3)
Period						
2005-2008	306 (25.3)	134 (23.4)	43 659 (29.5)	1979 (24.9)	702 (21.5)	272 521 (27.2)
2009-2012	423 (35.0)	204 (35.7)	50 613 (34.2)	2682 (33.7)	1048 (32.2)	329 736 (32.9)
2013-2016	479 (39.7)	234 (40.9)	53 538 (36.2)	3293 (41.4)	1509 (46.3)	400 464 (39.9)
Income, quartile^e^						
1st	448 (37.1)	251 (43.9)	62 417 (42.2)	3019 (38.0)	1340 (41.1)	391 121 (39.0)
2nd	466 (38.6)	261 (45.6)	46 629 (31.5)	2860 (36.0)	1542 (47.3)	322 330 (32.1)
3rd	172 (14.2)	43 (7.5)	20 700 (14.0)	1198 (15.1)	268 (8.2)	150 239 (15.0)
4th	122 (10.1)	17 (3.0)	18 064 (12.2)	877 (11.0)	109 (3.3)	139 031 (13.9)
Education level, y^f^						
≤10	611 (50.6)	367 (64.2)	81 937 (55.4)	3993 (50.2)	1943 (59.6)	522 835 (52.1)
>10 to ≤15	387 (32.0)	157 (27.4)	49 167 (33.3)	2559 (32.2)	986 (30.3)	349 828 (34.9)
>15	210 (17.4)	48 (8.4)	16 706 (11.3)	1402 (17.6)	330 (10.1)	130 058 (13.0)
Migration history						
Danish born	1181 (97.8)	558 (97.6)	145 155 (98.2)	7790 (97.9)	3134 (96.2)	984 228 (98.2)
Western immigrant	21 (1.7)	5 (0.9)	1456 (1.0)	110 (1.4)	65 (2.0)	9979 (1.0)
Nonwestern immigrant	6 (0.5)	9 (1.6)	1199 (0.8)	54 (0.7)	60 (1.8)	8514 (0.8)
Marital status						
Unmarried	126 (10.4)	217 (37.9)	9344 (6.3)	799 (10.0)	1175 (36.1)	60 833 (6.1)
Married	423 (35.0)	94 (16.4)	70 736 (47.9)	3019 (38.0)	534 (16.4)	515 319 (51.4)
Divorced	302 (25.0)	175 (30.6)	17 448 (11.8)	1807 (22.7)	1088 (33.4)	115 942 (11.6)
Widowed	357 (29.6)	86 (15.0)	50 282 (34.0)	2329 (29.3)	462 (14.2)	310 627 (31.0)
Comorbidity						
Congestive heart failure	206 (17.1)	116 (20.3)	20 433 (13.8)	2136 (26.9)	1012 (31.1)	226 539 (22.6)
Hypertension	819 (67.8)	342 (59.8)	107 883 (73.0)	6540 (82.2)	2568 (78.8)	860 520 (85.8)
Diabetes	296 (24.5)	179 (31.3)	26 408 (17.9)	2164 (27.2)	1098 (33.7)	207 891 (20.7)
Stroke/thromboembolism	299 (24.8)	119 (20.8)	31 093 (21.0)	1965 (24.7)	813 (24.9)	214 078 (21.3)
Vascular disease	251 (20.8)	123 (21.5)	35 457 (24.0)	1803 (22.7)	712 (21.8)	253 702 (25.3)
Kidney disease	138 (11.4)	51 (8.9)	9372 (6.3)	870 (10.9)	320 (9.8)	62 110 (6.2)
Prior bleeding	308 (25.5)	140 (24.5)	30 035 (20.3)	2255 (28.4)	884 (27.1)	248 045 (24.7)
Liver disease	47 (3.9)	23 (4.0)	2757 (1.9)	299 (3.8)	164 (5.0)	19 368 (1.9)
Alcohol abuse	241 (20.0)	146 (25.5)	6221 (4.2)	1615 (20.3)	857 (26.3)	43 144 (4.3)
Other substance abuse	96 (7.9)	63 (11.0)	704 (0.5)	640 (8.0)	378 (11.6)	4689 (0.5)
Dementia	187 (15.5)	59 (10.3)	7598 (5.1)	1431 (18.0)	422 (12.9)	61 364 (6.1)
Schizophrenia	98 (8.1)	NA	572 (0.4)	568 (7.1)	NA	3259 (0.3)
Bipolar disorder	NA	98 (17.1)	1208 (0.8)	NA	568 (17.4)	7954 (0.8)
NSAID treatment	122 (10.1)	62 (10.8)	15 535 (10.5)	604 (7.6)	319 (9.8)	68 256 (6.8)
Antiplatelet treatment	532 (44.0)	240 (42.0)	69 798 (47.2)	3339 (42.0)	1391 (42.7)	397 814 (39.7)
Time since AF, mean (SD), y	NA	NA	NA	6.87 (5.43)	6.11 (4.89)	6.73 (5.34)
1	NA	NA	NA	1012 (12.7)	474 (14.5)	127 964 (12.8)
2	NA	NA	NA	874 (11.0)	410 (12.6)	113 946 (11.4)
3	NA	NA	NA	759 (9.5)	363 (11.1)	101 906 (10.2)
4	NA	NA	NA	689 (8.7)	302 (9.3)	90 915 (9.1)
5	NA	NA	NA	4620 (58.1)	1710 (52.5)	567 990 (56.6)

Abbreviations: AF, atrial fibrillation; NA, not applicable; NSAID, nonsteroidal anti-inflammatory drug.

^a^ Number of unique patients with bipolar disorder was 1810, corresponding to a mean of 4.4 entries per patient.

^b^ Number of unique patients with schizophrenia was 786, corresponding to a mean of 4.1 entries per patient.

^c^ Number of unique patients was 199 219, corresponding to a mean of 5.0 entries per patient. The cohorts of incident and prevalent patients were only partially coinciding, because patients who had incident events before 2005 could be included in the prevalent group, whereas some patients who were incident in the inclusion period from 2005 through September 2016 could be censored due to emigration, end of follow-up, death, or other criteria before their first annual entry in the prevalent group.

^d^ Of unique patients in the prevalence group, 96 606 (105 with bipolar disorder and 441 with schizophrenia) were women; 102 613 (753 with bipolar disorder and 345 with schizophrenia) were men.

^e^ Year-specific quartiles of the gross Danish population.

^f^ Of unique patients in the prevalence group, 195 540 (1774 with bipolar disorder and 760 with schizophrenia) were Danish-born, 2043 (25 with bipolar disorder and 10 with schizophrenia) were Western immigrants, and 1636 (11 with bipolar disorder and 16 with schizophrenia) were nonwestern immigrants.

### Statistical Analysis

To assess the extent to which a possible OAT deficit in patients with bipolar disorder or schizophrenia could be associated with other characteristics of these patients, the OAT deficit was investigated under several different levels of adjustment. All adjusted analyses were performed on the entire cohort of all incident (for OAT initiation) or prevalent (for OAT prevalence) AF cases at increased risk. Selected, crude comparisons within this cohort were performed between patients with bipolar disorder or schizophrenia and a 1:3-matched random sample of patients without the respective diagnoses. For the initiation study, matching criteria included age, sex, CHA_2_DS_2_-VASc score, and calendar year. For the prevalence study, we also included years since AF diagnosis.

Baseline characteristics were described in the examination of both initiation and prevalence for the entire cohort, the subgroups with bipolar disorder and schizophrenia, and the matched random samples. For the prevalence study, this description was performed in terms of entry years (ie, patient data were weighted according to years of study eligibility).

To obtain a crude description of treatment initiation in patients with bipolar disorder or schizophrenia and the matched reference groups, we assessed their status 0 to 180 days after the index date, classified into initiated NOAC therapy, initiated vitamin K antagonist therapy, fulfilled exclusion criteria, or death. We used the Aalen-Johansen approach for competing risks; thus, in this analysis, patients were classified according to their first observed event. Emigration (<0.1%) was treated as a censoring event.

We used the pseudo-observation approach^33^ for the adjusted analyses of OAT initiation, calculating pseudo-observations for the cumulative incidence of OAT initiation stratified by age group, sex, and calendar period. In this calculation, death, exclusion, and emigration were considered censoring events, implying that the obtained results estimated the intent to initiate treatment.

For both initiation and prevalence, adjusted proportion differences (aPDs) with corresponding 95% CIs were estimated in linear least-squares models with robust variance estimation. These models included index year and the above-mentioned covariates, including 3-knotted sex- and period-specific splines for age at index date. In addition, the models for treatment prevalence included a covariate representing time since AF diagnosis and accounted for the correlation between multiple entries of the same patient by cluster robust variance estimation with patient as the cluster unit.

All analyses were performed from January 1 to June 15, 2020, using Stata, version 15 (StataCorp LLC).

## Results

### Cohort Characteristics

For the study of treatment initiation, we identified 147 810 eligible patients with incident AF (eFigure in the Supplement) among whom mean (SD) age was 76.9 (10.1) years; 78 577 (53.2%) were women, 69 233 (46.8%) were men (Table 1), and mean (SD) CHA_2_DS_2_-VASc score was 3.73 (1.41) (eTable 4 in the Supplement). This cohort included 1208 (0.8%) patients with bipolar disorder and 572 (0.4%) patients with schizophrenia. A total of 199 219 patients were eligible for the study of OAT prevalence at least once in the study period (mean, 5.0 annual entries), of whom 1810 had bipolar disorder (mean, 4.4 entries) and 786 had schizophrenia (mean, 4.1 entries) (Table 1). Compared with their sex-, age- and CHA_2_DS_2_-VASc-matched references, individuals with bipolar disorder or schizophrenia were more often unmarried or divorced and had higher rates of comorbidities, such as diabetes, heart failure, and substance abuse (eTable 5 in the Supplement), at the relevant index dates for the studies of both initiation and prevalence.

### OAT Initiation

**Figure 1.**
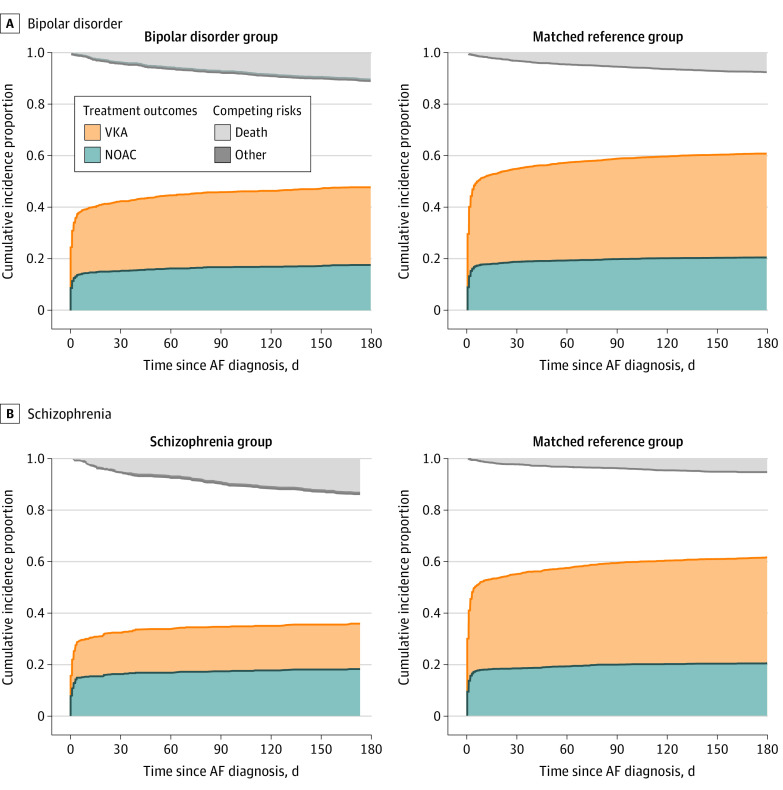
Cumulated Incidence of Oral Anticoagulation Initiation 0 to 180 Days After Incident Atrial Fibrillation (AF) Treatment status for patients with bipolar disorder (A) or schizophrenia (B) and for their matched reference groups when classified into initiated non–vitamin K antagonist oral anticoagulant (NOAC) therapy, initiated vitamin K antagonist (VKA) therapy, or the competing risks dead and fulfilling exclusion criteria according to first observed event. The reference groups were matched on sex, age, calendar year, and CHA_2_DS_2_-VASc (congestive heart failure, hypertension, age ≥75 years, diabetes, stroke or transient ischemic attack, vascular disease, age 65-74 years, sex category) score.

At initial hospital discharge with AF diagnosis, 24.5% (95% CI, 22.5%-26.6%) of patients with bipolar disorder vs 29.7% (95% CI, 28.4%-31.0%) of their matched referents, and 15.6% (95% CI, 13.5%-18.0%) of patients with schizophrenia vs 29.9% (95% CI, 28.0%-31.8%) were already receiving OAT. Ninety days post discharge, the proportions prescribed OAT increased to 46.0% (95% CI, 43.4%-48.5%) for patients with bipolar disorder vs 59.1% (95% CI, 57.5%-60.7%) for their matched referents and 34.6% (95% CI, 31.6%-37.8%) for patients with schizophrenia vs 59.4% (95% CI, 57.1%-61.7%) for their matched referents; NOAC therapy accounted for 16.8% (95% CI, 14.8%-19.0%) for patients with bipolar disorder vs 19.9% (95% CI, 18.6%-21.2%) for their matched referents and 17.3% (95% CI, 14.3%-20.5%) for those with schizophrenia vs 19.8% (95% CI, 18.0%-21.7%) for their matched referents. Most of this increase occurred within the first 2 weeks post discharge (Figure 1). The competing risk of death assessed 90 days post discharge was higher than among their matched referents both among patients with bipolar disorder (7.1%; 95% CI, 5.8%-8.7% vs 4.4%; 95% CI, 3.8%-5.1%) and those with schizophrenia (8.2%; 95% CI, 6.1%-10.6% vs 3.1%; 95% CI, 2.4%-4.0%) (Figure 1).

Still, the treated proportions were negligibly lower than the estimated intention to initiate OAT (46.5%; 95% CI, 43.7%-49.3% for bipolar disorder and 35.0%; 95% CI, 30.9%-39.1% for schizophrenia) used in the adjusted analyses. After adjustment for demographic characteristics, these figures corresponded to deficits in OAT initiation of 12.7% associated with bipolar disorder (aPD, −12.7%; 95% CI, −15.3% to −10.0%) and of 24.5% associated with schizophrenia (aPD, −24.5%; 95% CI, −28.3% to −20.7%). In the fully adjusted models, which also included socioeconomic characteristics and comorbidity, the estimated OAT initiation deficit associated with bipolar disorder was somewhat attenuated (aPD, −5.3%; 95% CI, −7.9% to −2.6%) (Table 2), but schizophrenia remained associated with a markedly lower likelihood of OAT initiation (aPD, −15.5%; 95% CI, −19.3% to −11.7%).

**Table 2.** Differences in OAT Initiation (Within 90 Days) and Overall OAT Prevalence Associated With Bipolar Disorder and Schizophrenia

Variable	Proportion, % (95% CI)	Adjusted proportion difference, % (95% CI)			
		Model 1	Model 2	Model 3	Model 4
		Demographic characteristics^a^	Model 1 with socioeconomic characteristics^b^	Model 2 with physical comorbidities^c^	Model 3 with psychiatric conditions^d^
**Initiation within 90 d of incident**					
Bipolar disorder	46.5 (43.7 to 49.3)	−12.7 (−15.3 to −10.0)	−11.7 (−14.4 to −9.0)	−10.6 (−13.2 to −7.9)	−5.3 (−7.9 to −2.6)
Schizophrenia	35.0 (30.9 to 39.1)	−24.5 (−28.3 to −20.7)	−21.7 (−25.5 to −17.9)	−20.1 (−23.9 to −16.3)	−15.5 (−19.3 to −11.7)
**Overall prevalence**					
Bipolar disorder	37.8 (36.7 to 38.9)	−11.6 (−13.9 to −9.3)	−11.0 (−13.3 to −8.7)	−9.4 (−11.4 to −7.3)	−4.9 (−7.0 to −2.9)
Schizophrenia	25.4 (23.7 to 27.1)	−21.6 (−24.8 to −18.4)	−20.0 (−23.2 to −16.8)	−17.2 (−20.2 to −14.1)	−12.8 (−15.9 to −9.7)

Abbreviation: OAT, oral anticoagulation therapy.

^a^ Adjusted for calendar year and sex- and period-specific 3-knotted cubic splines for age. Prevalence was additionally adjusted for years since atrial fibrillation diagnosis.

^b^ Additionally adjusted for income, educational level, migration history, and marital status.

^c^ Additionally adjusted for each of the CHA_2_DS_2_-VASc conditions, kidney disease, liver disease, prior bleeding event, antiplatelet treatment, and nonsteroidal anti-inflammatory drug treatment.

^d^ Additionally adjusted for history of alcohol abuse, other substance abuse, and dementia. Furthermore, the analyses of bipolar disorder were adjusted for schizophrenia and vice versa.

### OAT Prevalence

Among prevalent AF cases, 37.8% (95% CI, 36.7%-38.9%) of patients with bipolar disorder and 25.4% (95% CI, 23.7%-27.1%) of those with schizophrenia were prescribed OAT in the study period. These figures indicated significant OAT deficits when compared with the remaining patients after adjustment for demographics (bipolar disorder: aPD, −11.6%; 95% CI, −13.9% to −9.3%; schizophrenia: aPD, −21.6%; −24.8% to −18.4%) and even after full adjustment for socioeconomic characteristics and comorbidities (bipolar disorder: aPD, −4.9%; 95% CI, −7.0% to −2.9%; schizophrenia: aPD, −12.8%; 95% CI, −15.9% to −9.7%) (Table 2).

**Figure 2.**
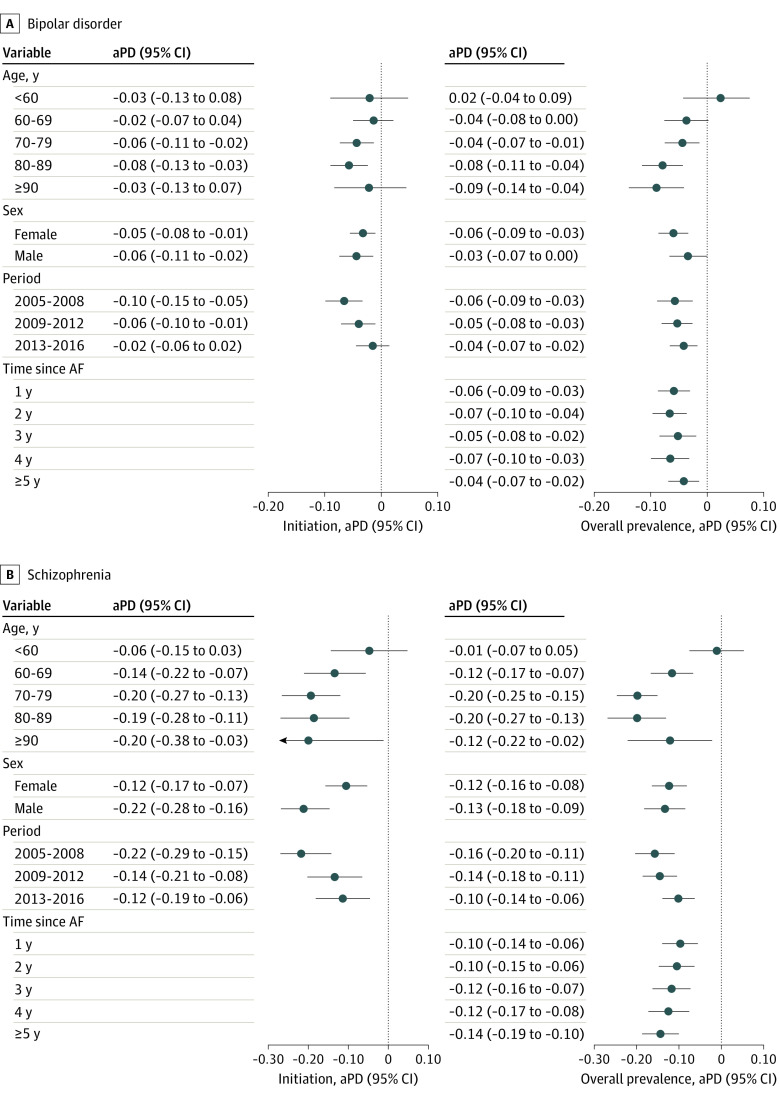
Associations Between Bipolar Disorder or Schizophrenia and Oral Anticoagulation Therapy (OAT) by Subgroup Fully adjusted proportion differences for OAT initiation within 90 days and overall OAT prevalence associated with bipolar disorder (A) and schizophrenia (B) in selected subgroups of patients with atrial fibrillation (AF) with increased risk status. aPD indicates adjusted proportion differences.

Figure 2 displays the aPDs with full adjustment for both OAT initiation and prevalence among subgroups of patients with comorbid AF and bipolar disorder or schizophrenia. The schizophrenia-associated deficits in OAT initiation and prevalence remained significant over time (aPD in 2013-2016 alone: −12.4%; 95% CI, −18.7% to −6.1% for initiation and −10.1%; 95% CI, −13.8% to −6.4% for prevalence), whereas this was not the case for the OAT initiation deficit associated with bipolar disorder (aPD in 2013-2016: −2.0%; 95% CI, −6.4% to 2.3%).

**Figure 3.**
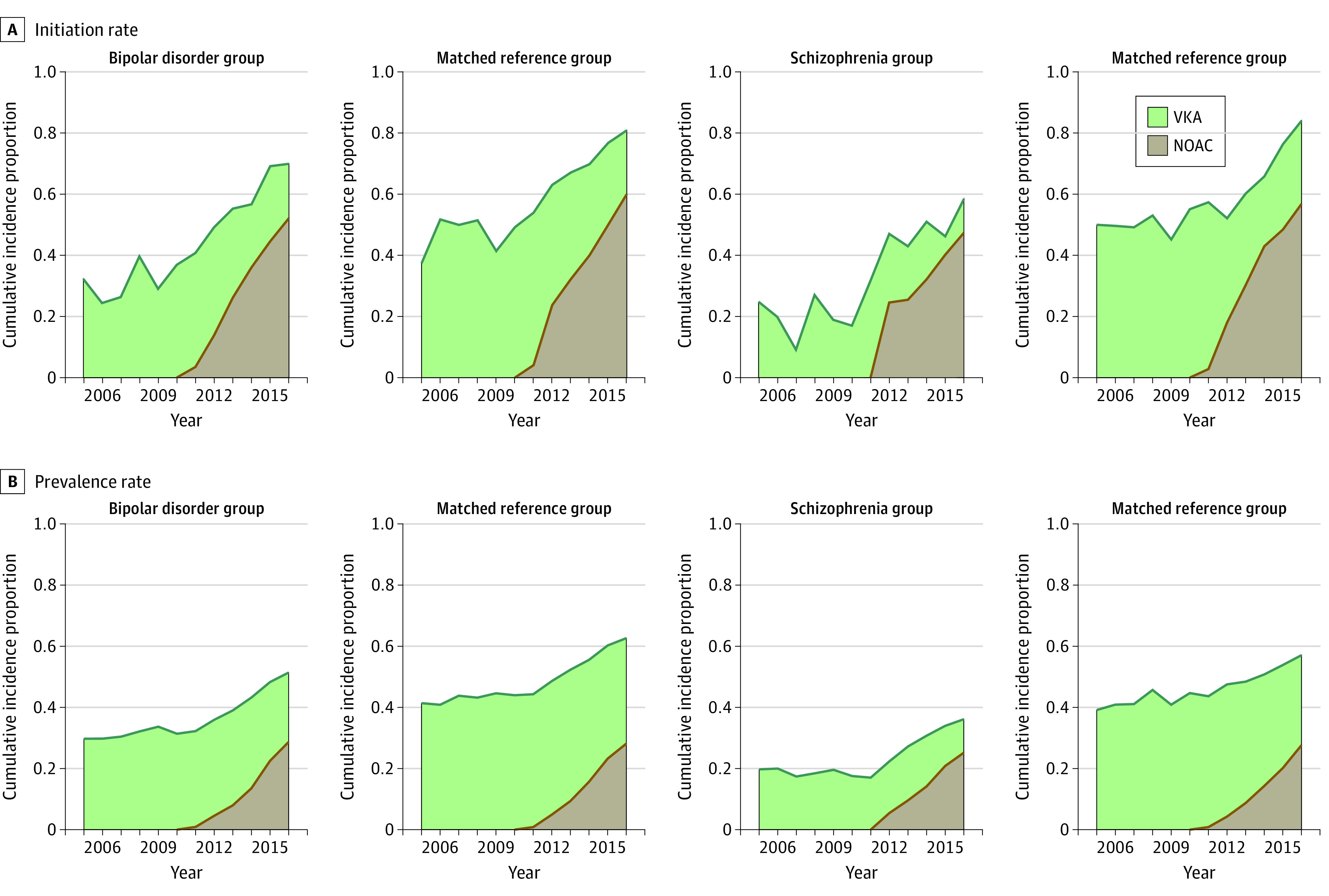
Temporal Development in Uptake of Oral Anticoagulation Therapy (OAT) Initiation (A) and overall prevalence (B) of OAT with either vitamin K antagonist (VKA) or non–vitamin K antagonist oral anticoagulant (NOAC) treatment in patients with atrial fibrillation and increased risk. Depicted values for OAT initiation were the pseudo-observation estimated intentions to initiate treatment within 90 days, which were also used in the adjusted analyses. Reference groups were matched on sex, age, calendar year, and CHA_2_DS_2_-VASc (congestive heart failure, hypertension, age ≥75 years, diabetes, stroke or transient ischemic attack, vascular disease, age 65-74 years, sex category) score. In analyses of treatment prevalence, the matching also included years since atrial fibrillation diagnosis.

With the introduction of NOAC therapy, a substantial increase in both OAT initiation and prevalence was observed for patients with comorbid bipolar disorder or schizophrenia (Figure 3). However, despite this change, significant deficits in overall OAT initiation and prevalence continued for those with comorbid schizophrenia. Sensitivity analyses with alternative durations of OAT redemptions showed no relevant impact on the results.

## Discussion

### Key Findings and Existing Literature

In this nationwide cohort study, bipolar disorder and schizophrenia were associated with significantly less OAT initiation within 90 days of hospitalization with AF and lower OAT prevalence in prevalent AF. For patients with bipolar disorder, the observed excess deficit in OAT initiation appeared to be largely associated with socioeconomic characteristics and additional comorbidity. However, this was not the case for individuals with schizophrenia, and their OAT deficit persisted even with the advent of NOAC.

The present study extends prior studies examining the influence of psychiatric comorbidity on use of OAT in AF. Previous studies lacked sufficient power to examine the association between a diagnosis of comorbid bipolar disorder or schizophrenia and receiving OAT,^25,26^ focused only on vitamin K antagonists,^25,26^ or were not specifically designed to address whether any deficits in use of OAT could represent a health care disparity beyond what could be related to relevant patient characteristics.^27^ Our findings are in keeping with smaller studies using Veterans Health Administration data, which found that any psychiatric diagnosis^25^ or a psychotic disorder diagnosis^26^ in veterans with AF was associated with lower likelihood of being prescribed a vitamin K antagonist.

Although patients with bipolar disorder or schizophrenia may have a greater number of medical comorbidities,^34,35^ to our knowledge, only one previous study included eligibility according to the CHA_2_DS_2_-VASc risk score.^26^ Moreover, extensive information on socioeconomic factors and comorbidity, including components of contraindication scores, allowed us to explore whether lower OAT uptake could be attributed to characteristics other than bipolar disorder or schizophrenia or was isolated to specific patient subgroups. In addition, to our knowledge, no studies have analyzed whether the introduction of NOAC therapy has affected the OAT uptake in patients with bipolar disorder or schizophrenia and AF.

### Interpretation and Perspectives of Findings

Suggestions for mechanisms underlying observed links between severe mental illness and development of, or worse outcomes associated with, cardiovascular diseases include autonomic nervous system dysfunction,^36^ systemic inflammation,^37^ shared risk genes,^38^ lifestyle,^39,40^ antipsychotic medications,^40^ and lower likelihood of receiving preventive and/or curative treatments.^41,42^ Our results suggest a potential contribution of the latter factor, particularly for individuals with schizophrenia. Therefore, it is reasonable to consider whether the observed deficit in OAT reflects poorer treatment adherence,^19,20^ clinically well-founded prescribing reticence (eg, due to comorbidities), or inequity in provided care.^41^ In our study, the rather constant OAT deficit among patients with bipolar disorder or schizophrenia over time since diagnosis does not indicate poor adherence. Hence, the lower treatment initiation appears to be the primary source of the OAT deficit in these patients. That this deficit was found to exceed what could be related to patient characteristics suggests a health disparity particularly affecting individuals with schizophrenia.

Yet, there may be clinically valid reasons not captured in our data for OAT not being prescribed for patients with bipolar disorder or schizophrenia. One concern could be increased risk of bleeding with OAT in this population,^43^ possibly related to increased alcohol use,^44,45,46^ poorer anticoagulation control for patients receiving a vitamin K antagonist,^43^ and greater likelihood of receiving antidepressants with anticoagulant effects.^47,48,49^ Alcohol abuse was more common among patients with bipolar disorder or schizophrenia vs the matched reference groups, and although the fully adjusted models accounted for this factor, we cannot discern the potential relevance of alcohol use below the diagnosis threshold.

However, one population-based study suggested that individuals with comorbid bipolar disorder or schizophrenia and AF were not at a substantially increased risk of major bleeding, and adjusting for OAT did not appear to affect this finding.^27^ Furthermore, even if concerns about vitamin K antagonist treatment in patients with comorbid psychiatric illnesses and AF remain, the availability of NOACs may minimize clinicians’ reticence to prescribe OAT. Although we found that patients with comorbid AF and bipolar disorder or schizophrenia were increasingly likely to receive NOACs over time, a deficit in the use of OAT persisted in individuals with comorbid schizophrenia. In addition, we found that patients with schizophrenia were more likely than the reference group to die following their index hospitalization with AF before initiating OAT.

Moreover, schizophrenia has been associated with a higher likelihood of having undiagnosed AF.^50^ Therefore, we may have underestimated the disparity in patients with comorbid schizophrenia and AF receiving OAT.

Our findings add to prior work^27^ calling for further research investigating whether interventions to enhance organized care for patients with comorbid schizophrenia and AF could increase OAT prescription and adherence and lead to reduced risk of stroke or other adverse events. Studies have suggested benefits of interventions aiming to reduce cardiometabolic risk factors and improve physical health-related quality of life in patients with severe mental illnesses,^51,52,53^ which holds a promise for patients with comorbid schizophrenia and AF.

### Strengths and Limitations

The nationwide registry data used have several strengths, including low risk of selection and recall biases owing to prospective and near complete registration of valid AF diagnoses^29^ and OAT prescriptions, along with virtually no loss to follow-up. However, limitations remain.

We lacked exact information on OAT duration for each prescription. However, our sensitivity analyses suggested that changing the grace period for OAT prescription affected results negligibly. Furthermore, although the reliability of psychiatric diagnoses in the registry data are high,^54^ individuals with these illnesses who did not present for treatment could be missed.

Similarly, hypertension, which is both an indication for OAT (based on the CHA_2_DS_2_-VASc score) and a potential contraindication (based on the HAS-BLED score), is often detected and handled in primary care, and diagnoses from this setting are not recorded in the registries. However, this limitation is likely to be minimal in the present study population because hypertension is the most common comorbidity with AF^55^ and so would likely have been captured in hospital contacts. Furthermore, the condition was also identified from prescription data. Moreover, we lacked data on labile international normalized ratios, a component of the HAS-BLED. Yet, the clinical relevance of this limitation is unclear because this information is generally not available before OAT initiation and is not applicable for NOAC treatment.^56^ In addition, although our analyses attempted to minimize this possibility, the potential of residual confounding remains, as with any observational study.

## Conclusions

In this study, comorbid bipolar disorder and schizophrenia appear to be associated with less OAT initiation in patients with incident AF and lower OAT prevalence in patients with prevalent AF. Although these associations appear primarily to be related to socioeconomic factors and comorbidities in patients with bipolar disorder, the associations are independent of these characteristics in patients with comorbid schizophrenia and AF. These findings suggest that individuals with comorbid schizophrenia and AF are less likely to receive evidence-based OAT vs those without severe mental illness. Research is needed to examine whether improved care for individuals with severe mental illnesses and comorbid AF reduces this health care disparity.

## Notes

Supplement. **eMethods.** Data Sources eReferences **eFigure 1.** Flow Chart for the Construction of the Cohort of Incident AF Patients **eTable 1.** Specification of Definition of the CHA_2_DS_2_-VASc Risk Score **eTable 2.** Specification of Exclusion Criteria **eTable 3.** Specification of Additional Comorbidities and Treatment Included in the Adjustments **eTable 4.** Distribution of CHA_2_DS_2_-VASc Scores in the Study Cohorts and the Subgroups With Bipolar Disorder or Schizophrenia **eTable 5.** Characteristics of Matched References for Those With Bipolar Disorder or Schizophrenia

## References

[1] Walker ER, McGee RE, Druss BG. Mortality in mental disorders and global disease burden implications: a systematic review and meta-analysis. JAMA Psychiatry. 2015;72(4):334-341. doi:10.1001/jamapsychiatry.2014.2502 25671328 PMC4461039

[2] Erlangsen A, Andersen PK, Toender A, Laursen TM, Nordentoft M, Canudas-Romo V. Cause-specific life-years lost in people with mental disorders: a nationwide, register-based cohort study. Lancet Psychiatry. 2017;4(12):937-945. doi:10.1016/S2215-0366(17)30429-7 29122573

[3] Laursen TM, Wahlbeck K, Hällgren J, . Life expectancy and death by diseases of the circulatory system in patients with bipolar disorder or schizophrenia in the Nordic countries. PLoS One. 2013;8(6):e67133. doi:10.1371/journal.pone.0067133 23826212 PMC3691116

[4] Ösby U, Westman J, Hällgren J, Gissler M. Mortality trends in cardiovascular causes in schizophrenia, bipolar and unipolar mood disorder in Sweden 1987-2010. Eur J Public Health. 2016;26(5):867-871. doi:10.1093/eurpub/ckv245 26748100 PMC5054269

[5] Patel NJ, Deshmukh A, Pant S, . Contemporary trends of hospitalization for atrial fibrillation in the United States, 2000 through 2010: implications for healthcare planning. Circulation. 2014;129(23):2371-2379. doi:10.1161/CIRCULATIONAHA.114.008201 24842943

[6] Benjamin EJ, Virani SS, Callaway CW, ; American Heart Association Council on Epidemiology and Prevention Statistics Committee and Stroke Statistics Subcommittee. heart disease and stroke statistics—2018 update: a report from the American Heart Association. Circulation. 2018;137(12):e67-e492. doi:10.1161/CIR.0000000000000558 29386200

[7] Staerk L, Wang B, Preis SR, . Lifetime risk of atrial fibrillation according to optimal, borderline, or elevated levels of risk factors: cohort study based on longitudinal data from the Framingham Heart Study. BMJ. 2018;361:k1453. doi:10.1136/bmj.k1453 29699974 PMC5917175

[8] Dorian P, Jung W, Newman D, . The impairment of health-related quality of life in patients with intermittent atrial fibrillation: implications for the assessment of investigational therapy. J Am Coll Cardiol. 2000;36(4):1303-1309. doi:10.1016/S0735-1097(00)00886-X 11028487

[9] Shinbane JS, Wood MA, Jensen DN, Ellenbogen KA, Fitzpatrick AP, Scheinman MM. Tachycardia-induced cardiomyopathy: a review of animal models and clinical studies. J Am Coll Cardiol. 1997;29(4):709-715. doi:10.1016/S0735-1097(96)00592-X 9091514

[10] Santangeli P, Di Biase L, Bai R, . Atrial fibrillation and the risk of incident dementia: a meta-analysis. Heart Rhythm. 2012;9(11):1761-1768. doi:10.1016/j.hrthm.2012.07.026 22863685

[11] Saglietto A, Matta M, Gaita F, Jacobs V, Bunch TJ, Anselmino M. Stroke-independent contribution of atrial fibrillation to dementia: a meta-analysis. Open Heart. 2019;6(1):e000984. doi:10.1136/openhrt-2018-000984 31217998 PMC6546265

[12] Wolf PA, Abbott RD, Kannel WB. Atrial fibrillation as an independent risk factor for stroke: the Framingham Study. Stroke. 1991;22(8):983-988. doi:10.1161/01.STR.22.8.983 1866765

[13] Johnson W, Onuma O, Owolabi M, Sachdev S. Stroke: a global response is needed. Bull World Health Organ. 2016;94(9):634-634A. doi:10.2471/BLT.16.181636 27708464 PMC5034645

[14] Hart RG, Pearce LA, Aguilar MI. Meta-analysis: antithrombotic therapy to prevent stroke in patients who have nonvalvular atrial fibrillation. Ann Intern Med. 2007;146(12):857-867. doi:10.7326/0003-4819-146-12-200706190-00007 17577005

[15] Ruff CT, Giugliano RP, Braunwald E, . Comparison of the efficacy and safety of new oral anticoagulants with warfarin in patients with atrial fibrillation: a meta-analysis of randomised trials. Lancet. 2014;383(9921):955-962. doi:10.1016/S0140-6736(13)62343-0 24315724

[16] Kirchhof P, Benussi S, Kotecha D, ; ESC Scientific Document Group. 2016 ESC guidelines for the management of atrial fibrillation developed in collaboration with EACTS. Eur Heart J. 2016;37(38):2893-2962. doi:10.1093/eurheartj/ehw210 27567408

[17] January CT, Wann LS, Calkins H, . 2019 AHA/ACC/HRS focused update of the 2014 AHA/ACC/HRS Guideline for the management of patients with atrial fibrillation: a report of the American College of Cardiology/American Heart Association task force on clinical practice guidelines and the Heart Rhythm Society. J Am Coll Cardiol. 2019;74(1):104-132. doi:10.1016/j.jacc.2019.01.011 30703431

[18] Gadsbøll K, Staerk L, Fosbøl EL, . Increased use of oral anticoagulants in patients with atrial fibrillation: temporal trends from 2005 to 2015 in Denmark. Eur Heart J. 2017;38(12):899-906. doi:10.1093/eurheartj/ehw658 28110293

[19] Piette JD, Heisler M, Ganoczy D, McCarthy JF, Valenstein M. Differential medication adherence among patients with schizophrenia and comorbid diabetes and hypertension. Psychiatr Serv. 2007;58(2):207-212. doi:10.1176/ps.2007.58.2.207 17287377

[20] Levin JB, Aebi ME, Tatsuoka C, Cassidy KA, Sajatovic M. Adherence to psychotropic and nonpsychotropic medication among patients with bipolar disorder and general medical conditions. Psychiatr Serv. 2016;67(3):342-345. doi:10.1176/appi.ps.201500010 26695494 PMC4934383

[21] Kilbourne AM, Goodrich DE, Lai Z, . Randomized controlled trial to assess reduction of cardiovascular disease risk in patients with bipolar disorder: the Self-Management Addressing Heart Risk Trial (SMAHRT). J Clin Psychiatry. 2013;74(7):e655-e662. doi:10.4088/JCP.12m08082 23945460 PMC4154058

[22] McKibbin CL, Lee A, Glaser D, . Functional health status of adults with serious mental illness and diabetes mellitus: a latent profile analysis. Int J Psychiatry Med. 2019;54(1):22-38. doi:10.1177/0091217418791437 30079813

[23] Tsai KY, Lee CC, Chou YM, Su CY, Chou FH. The incidence and relative risk of stroke in patients with schizophrenia: a five-year follow-up study. Schizophr Res. 2012;138(1):41-47. doi:10.1016/j.schres.2012.02.013 22386734

[24] Prieto ML, Cuéllar-Barboza AB, Bobo WV, . Risk of myocardial infarction and stroke in bipolar disorder: a systematic review and exploratory meta-analysis. Acta Psychiatr Scand. 2014;130(5):342-353. doi:10.1111/acps.12293 24850482 PMC5023016

[25] Walker GA, Heidenreich PA, Phibbs CS, . Mental illness and warfarin use in atrial fibrillation. Am J Manag Care. 2011;17(9):617-624. 21902447

[26] Schmitt SK, Turakhia MP, Phibbs CS, . Anticoagulation in atrial fibrillation: impact of mental illness. Am J Manag Care. 2015;21(11):e609-e617. 26735294

[27] Søgaard M, Skjøth F, Kjældgaard JN, Larsen TB, Hjortshøj SP, Riahi S. Atrial fibrillation in patients with severe mental disorders and the risk of stroke, fatal thromboembolic events and bleeding: a nationwide cohort study. BMJ Open. 2017;7(12):e018209. doi:10.1136/bmjopen-2017-018209 29217725 PMC5728273

[28] Capewell S, Graham H. Will cardiovascular disease prevention widen health inequalities? PLoS Med. 2010;7(8):e1000320. doi:10.1371/journal.pmed.1000320 20811492 PMC2927551

[29] Rix TA, Riahi S, Overvad K, Lundbye-Christensen S, Schmidt EB, Joensen AM. Validity of the diagnoses atrial fibrillation and atrial flutter in a Danish patient registry. Scand Cardiovasc J. 2012;46(3):149-153. doi:10.3109/14017431.2012.673728 22397620

[30] Christesen AMS, Vinter N, Mortensen LS, Fenger-Grøn M, Johnsen SP, Frost L. Inequality in oral anticoagulation use and clinical outcomes in atrial fibrillation: a Danish nationwide perspective. Eur Heart J Qual Care Clin Outcomes. 2018;4(3):189-199. doi:10.1093/ehjqcco/qcy011 30102321

[31] Ribe AR, Vestergaard CH, Vestergaard M, . Statins and risk of intracerebral hemorrhage in individuals with a history of stroke. Stroke. 2020;51(4):1111-1119. doi:10.1161/STROKEAHA.119.027301 32114928

[32] Fenger-Grøn M, Vestergaard CH, Frost L, . Depression and uptake of oral anticoagulation therapy in patients with atrial fibrillation: a Danish nationwide cohort study. Med Care. 2020;58(3):216-224. doi:10.1097/MLR.0000000000001268 31876644

[33] Klein JP, Andersen PK. Regression modeling of competing risks data based on pseudovalues of the cumulative incidence function. Biometrics. 2005;61(1):223-229. doi:10.1111/j.0006-341X.2005.031209.x 15737097

[34] Carney CP, Jones LE. Medical comorbidity in women and men with bipolar disorders: a population-based controlled study. Psychosom Med. 2006;68(5):684-691. doi:10.1097/01.psy.0000237316.09601.88 17012521

[35] Carney CP, Jones L, Woolson RF. Medical comorbidity in women and men with schizophrenia: a population-based controlled study. J Gen Intern Med. 2006;21(11):1133-1137. doi:10.1111/j.1525-1497.2006.00563.x 17026726 PMC1831667

[36] Quintana DS, Westlye LT, Kaufmann T, . Reduced heart rate variability in schizophrenia and bipolar disorder compared to healthy controls. Acta Psychiatr Scand. 2016;133(1):44-52. doi:10.1111/acps.12498 26371411

[37] Chung KH, Chen PH, Kuo CJ, Tsai SY, Huang SH, Wu WC. Risk factors for early circulatory mortality in patients with schizophrenia. Psychiatry Res. 2018;267:7-11. doi:10.1016/j.psychres.2018.05.044 29879603

[38] Mäki-Marttunen T, Lines GT, Edwards AG, . Pleiotropic effects of schizophrenia-associated genetic variants in neuron firing and cardiac pacemaking revealed by computational modeling. Transl Psychiatry. 2017;7(11):5. doi:10.1038/s41398-017-0007-4 30446648 PMC5802468

[39] Gardner-Sood P, Lally J, Smith S, ; IMPaCT team. Cardiovascular risk factors and metabolic syndrome in people with established psychotic illnesses: baseline data from the IMPaCT randomized controlled trial. Psychol Med. 2015;45(12):2619-2629. doi:10.1017/S0033291715000562 25961431 PMC4531468

[40] Correll CU, Robinson DG, Schooler NR, . Cardiometabolic risk in patients with first-episode schizophrenia spectrum disorders: baseline results from the RAISE-ETP study. JAMA Psychiatry. 2014;71(12):1350-1363. doi:10.1001/jamapsychiatry.2014.1314 25321337

[41] DE Hert M, Correll CU, Bobes J, . Physical illness in patients with severe mental disorders—I: prevalence, impact of medications and disparities in health care. World Psychiatry. 2011;10(1):52-77. doi:10.1002/j.2051-5545.2011.tb00014.x 21379357 PMC3048500

[42] Bongiorno DM, Daumit GL, Gottesman RF, Faigle R. Comorbid psychiatric disease Is associated with lower rates of thrombolysis in ischemic stroke. Stroke. 2018;49(3):738-740. doi:10.1161/STROKEAHA.117.020295 29374106 PMC5829001

[43] Paradise HT, Berlowitz DR, Ozonoff A, . Outcomes of anticoagulation therapy in patients with mental health conditions. J Gen Intern Med. 2014;29(6):855-861. doi:10.1007/s11606-014-2784-2 24549520 PMC4026501

[44] Koskinen J, Löhönen J, Koponen H, Isohanni M, Miettunen J. Prevalence of alcohol use disorders in schizophrenia—a systematic review and meta-analysis. Acta Psychiatr Scand. 2009;120(2):85-96. doi:10.1111/j.1600-0447.2009.01385.x 19374633

[45] Farren CK, Hill KP, Weiss RD. Bipolar disorder and alcohol use disorder: a review. Curr Psychiatry Rep. 2012;14(6):659-666. doi:10.1007/s11920-012-0320-9 22983943 PMC3730445

[46] Roth JA, Bradley K, Thummel KE, Veenstra DL, Boudreau D. Alcohol misuse, genetics, and major bleeding among warfarin therapy patients in a community setting. Pharmacoepidemiol Drug Saf. 2015;24(6):619-627. doi:10.1002/pds.3769 25858232 PMC4478047

[47] Himelhoch S, Slade E, Kreyenbuhl J, Medoff D, Brown C, Dixon L. Antidepressant prescribing patterns among VA patients with schizophrenia. Schizophr Res. 2012;136(1-3):32-35. doi:10.1016/j.schres.2012.01.008 22325077

[48] Siddiqui R, Gawande S, Shende T, Tadke R, Bhave S, Kirpekar V. SSRI-induced coagulopathy: is it reality? Ther Adv Psychopharmacol. 2011;1(6):169-174. doi:10.1177/2045125311423781 23983943 PMC3736913

[49] Quinn GR, Singer DE, Chang Y, . Effect of selective serotonin reuptake inhibitors on bleeding risk in patients with atrial fibrillation taking warfarin. Am J Cardiol. 2014;114(4):583-586. doi:10.1016/j.amjcard.2014.05.037 25001151 PMC5176251

[50] Smith DJ, Langan J, McLean G, Guthrie B, Mercer SW. Schizophrenia is associated with excess multiple physical-health comorbidities but low levels of recorded cardiovascular disease in primary care: cross-sectional study. BMJ Open. 2013;3(4):e002808. doi:10.1136/bmjopen-2013-002808 23599376 PMC3641427

[51] Druss BG, von Esenwein SA, Compton MT, Rask KJ, Zhao L, Parker RM. A randomized trial of medical care management for community mental health settings: the Primary Care Access, Referral, and Evaluation (PCARE) study. Am J Psychiatry. 2010;167(2):151-159. doi:10.1176/appi.ajp.2009.09050691 20008945 PMC3775666

[52] Kilbourne AM, Barbaresso MM, Lai Z, . Improving physical health in patients with chronic mental disorders: twelve-month results from a randomized controlled collaborative care trial. J Clin Psychiatry. 2017;78(1):129-137. doi:10.4088/JCP.15m10301 27780336 PMC5272777

[53] Chwastiak LA, Luongo M, Russo J, . Use of a mental health center collaborative care team to improve diabetes care and outcomes for patients with psychosis. Psychiatr Serv. 2018;69(3):349-352. doi:10.1176/appi.ps.201700153 29191136

[54] Jakobsen KD, Frederiksen JN, Hansen T, Jansson LB, Parnas J, Werge T. Reliability of clinical ICD-10 schizophrenia diagnoses. Nord J Psychiatry. 2005;59(3):209-212. doi:10.1080/08039480510027698 16195122

[55] Seccia TM, Caroccia B, Muiesan ML, Rossi GP. Atrial fibrillation and arterial hypertension: a common duet with dangerous consequences where the renin angiotensin-aldosterone system plays an important role. Int J Cardiol. 2016;206:71-76. doi:10.1016/j.ijcard.2016.01.007 26774837

[56] Singer DE. Methodologic problems in the assessment of bleed scores. J Am Coll Cardiol. 2013;61(4):481. doi:10.1016/j.jacc.2012.09.052 23347783

