# Supplementary material for: Association Between Bipolar Disorder or Schizophrenia and Oral Anticoagulation Use in Danish Adults With Incident or Prevalent Atrial Fibrillation
Source: JAMA Netw Open. 2021 May 17;4(5):e2110096. doi: 10.1001/jamanetworkopen.2021.10096 (PMC8129823; doi:10.1001/jamanetworkopen.2021.10096)
Supplement: Supplement. — eMethods. Data Sources eReferences eFigure 1. Flow Chart for the Construction of the Cohort of Incident AF Patients eTable 1. Specification of Definition of the CHA2DS2-VASc Risk Score eTable 2. Specification of Exclusion Criteria eTable 3. Specification of Additional Comorbidities and Treatment Included in the Adjustments eTable 4. Distribution of CHA2DS2-VASc Scores in the Study Cohorts and the Subgroups With Bipolar Disorder or Schizophrenia eTable 5. Characteristics of Matched References for Those With Bipolar Disorder or Schizophrenia [file jamanetwopen-e2110096-s001.pdf]

**Supplementary Online Content**

Fenger-Grøn M, Vestergaard CH, Ribe AR, et al. Association between bipolar disorder or schizophrenia and oral anticoagulation use in Danish adults with incident or prevalent atrial fibrillation. *JAMA Netw Open*. 2021;4(5):e2110096. doi:10.1001/jamanetworkopen.2021.10096

**eMethods.** Data Sources

**eReferences**

**eFigure 1.** Flow Chart for the Construction of the Cohort of Incident AF Patients

**eTable 1.** Specification of Definition of the $CHA_2DS_2$-VASc Risk Score

**eTable 2.** Specification of Exclusion Criteria

**eTable 3.** Specification of Additional Comorbidities and Treatment Included in the Adjustments

**eTable 4.** Distribution of $CHA_2DS_2$-VASc Scores in the Study Cohorts and the Subgroups With Bipolar Disorder or Schizophrenia

**eTable 5.** Characteristics of Matched References for Those With Bipolar Disorder or Schizophrenia

This supplementary material has been provided by the authors to give readers additional information about their work.

© 2021 Fenger-Grøn M et al. *JAMA Network Open.*

*eMethods. Data sources*

Data on hospital diagnoses and procedures was obtained from the Danish National Patient Register.[1] This register includes dates and primary/secondary discharge diagnoses for admissions to non-psychiatric hospitals in Denmark since 1977 as well as outpatient contacts, emergency room contacts, and all contacts to psychiatric hospitals since 1995. Until 1993, diagnostic classification was made according to the Danish version of the International Classification of Diseases, 8th Revision (ICD-8) and subsequently according to the ICD-10.

Medication data was obtained from the Danish National Prescription Registry, which contains information on all prescriptions redeemed at Danish pharmacies since 1995.[2]

Data on age, sex, death, and migration was collected from the Danish Civil Registration System.[3] A feature of this register is the unique Danish civil registration number, which is assigned to all Danish citizens at birth or emigration. This number enables individual-level linkage between all of the above-mentioned registries and databases at Statistics Denmark, which holds data on socioeconomic characteristics.

**eReferences**


1. Schmidt M, Schmidt SA, Sandegaard JL, Ehrenstein V, Pedersen L, Sorensen HT. The Danish National Patient Registry: a review of content, data quality, and research potential. *Clin Epidemiol.* 2015;7:449-490.

2. Kildemoes HW, Sorensen HT, Hallas J. The Danish National Prescription Registry. *Scand J Public Health.* 2011;39(7 Suppl):38-41.

3. Pedersen CB. The Danish Civil Registration System. *Scand J Public Health.* 2011;39(7 Suppl):22-25.


© 2021 Fenger-Grøn M et al. *JAMA Network Open.*

eFigure 1: Flow chart for the construction of the cohort of incident AF patients (for assessment of OAT initiation):

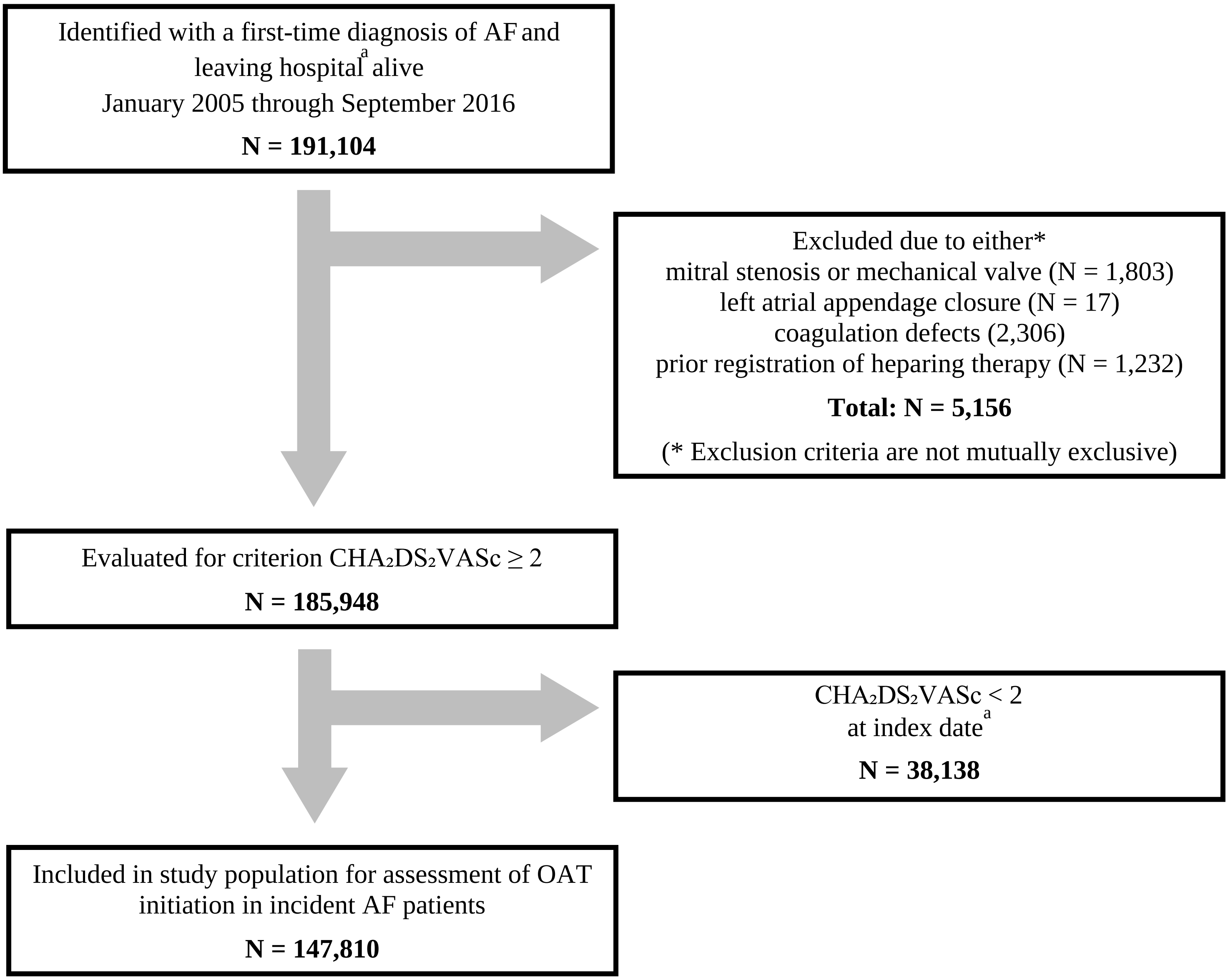


[a] For outpatients courses with a duration of more than one day, the date of 'leaving hospital with an AF diagnosis' (i.e. the index date for the study) was set to the date of the first visit. For inpatient contacts, the date of leaving hospital was set to the date of discharge unless the discharge was followed by a new hospital admission on the same day. In this case, the discharge registration was assumed to represent a transferral between departments, and the date of discharge from the last of the subsequent consecutive admissions was used.

© 2021 Fenger-Grøn M et al. *JAMA Network Open.*

eTable 1. Specification of definition of the $CHA_2DS_2$-VASc risk score.
The risk score assesses the risk of thromboembolic event based on demographic characteristics: female sex (1 point), age 65-74 years (1 point), age ≥ 75 years (2 points), and the below-mentioned comorbidities. All characteristics were assessed on the relevant index dates, ie, the date of AF diagnosis for the OAT initiation study and annually after this date for the OAT prevalence study.

| | | | |
|---|---|---|---|
| **Congestive heart failure (1 point)** | | Diagnosis AND at least one prescription of loop diuretics (from 1 year before the AF diagnosis or at any later point) | |
| | | Diagnosis | ICD-10: I11.0; I13.0; I13.2; I42.0; I50 |
| | Loop diuretics | Prescription | ATC: C03C |
| **Hypertension (1 point)** | | Diagnosis AND/OR treatment with at least two classes of antihypertensive drugs within 1 year (from 1 year before the AF diagnosis until end of follow-up) | |
| | | Diagnosis | ICD-10: I10; I11; I12; I13; I15 |
| | α adrenergic blockers | Prescription | ATC: C02A; C02B; C02C |
| | Non-loop diuretics | Prescription | ATC: C02DA; C02L; C03A; C03B; C03D; C03E; C03X; C07B; C07C; C07D; C08G; C09BA; C09DA; C09XA52 |
| | Vasodilators | Prescription | ATC: C02DB; C02DD; C02DG; C04; C05 |
| | β blockers | Prescription | ATC: C07 |
| | Calcium channel blockers | Prescription | ATC: C08; C09BB; C09DB |
| | Renin-angiotensin system inhibitors | Prescription | ATC: C09 |
| **Diabetes mellitus (1 point)** | | Diagnosis AND/OR treatment with antidiabetics | |
| | | Diagnosis | ICD-10: E10; E11; E12; E13; E14; H360 |
| | | Prescription | ATC: A10A; A10B |
| **Previous thromboembolism (2 points)** | | Diagnosis of ischemic stroke, transient ischemic attack (TIA), or systemic embolism | |
| | Ischemic stroke | Diagnosis | ICD-10: I63 (except I636 due to the venous origin); I64 |
| | Transient ischemic attack (TIA) | Diagnosis | ICD-10: G45 (except G453 and G454) |
| | Systemic embolism | Diagnosis | ICD-10: I74; K550; N280 |
| **Vascular disease (1 point)** | | Diagnosis of myocardial infarction or peripheral artery disease (PAD) or registration of a coronary procedure | |
| | Myocardial infarction | Diagnosis | ICD-10: I21; I22 |
| | Peripheral artery disease (PAD) | Diagnosis | ICD-10: I65; I70 |
| | Coronary procedure | Procedure | NCSP: FN |

© 2021 Fenger-Grøn M et al. *JAMA Network Open.*

eTable 2: Specification of exclusion criteria.

| | | |
|---|---|---|
| **Mitral stenosis** (diagnosis or procedure) | Diagnosis | ICD-10: I050; I052; I342 |
| | Procedure | FKA |
| **Mechanical prosthetic heart valves** | Procedure | FCA60; FGE00; FJF00; FKD00; FMD00 |
| **Left atrial appendage closure** | Procedure | FFW98A |
| **Coagulation defects** | Diagnosis | ICD-10: D66-D69 |
| **Heparin treatment**[a] | Prescription | ATC: B01AB |

[a] Patients receiving heparin therapy were not considered eligible for OAT. As information on medical treatment was obtained from the Danish National Prescription Registry, hospital-administered heparin treatment during the patients' initial inpatient course did not entail exclusion.

© 2021 Fenger-Grøn M et al. *JAMA Network Open.*

eTable 3: Specification of additional comorbidities and treatment included in the adjustments. All characteristics were assessed on the relevant index dates, ie, the date of AF diagnosis for the OAT initiation study and annually after this date for the OAT prevalence study.

| | | |
|---|---|---|
| **Kidney disease** | Diagnosis | ICD-10: E102; E112; E132; E142; I12; I13; N03; N04; N05; N07; N08; N11; N14; N158; N159; N16; N18 (except N181 and N182); N19; N26; Q61 |
| **Liver disease** | Diagnosis | ICD-10: B18; C22; D684C; K70; K71 (except K710; K711A; K711B; K712 and K716); K72; K73; K74; K75; K76; K77; Q618A; Z944 |
| **Prior bleeding incidence** | Diagnosis | ICD-10: I60, I61; I62 (intracranial bleeding) |
| | Diagnosis | ICD-10: D500; D62 I850; I864A K250; K252; K254; K256; K260; K262; K264; K266; K270; K272; K274; K276; K280; K282; K284; K286 K290; K298A; K625; K920; K921; K922 J942; R04 N02; R31 R58 (other major bleeding) |
| **Alcohol abuse** | Diagnosis | ICD-10: E244; E512; E52; F10; G312; G621; G721; I426; K292; K70; K860; L278A; R780; T51; Z502; Z714; Z721; K852; T500A |
| **Other substance abuse** | Diagnosis | ICD-10: F11-F16; F18-F19 |
| **Dementia** | Diagnosis | ICD-10: F00-F03; F051; G30 |
| (diagnosis and/or prescription) | Prescription | ATC: N06D |
| **Antiplatelet treatment**[a] | Prescription | ATC: B01AC04; N02BA01; B01AC06; B01AC07; B01AC22; B01AC24; B01AC30 |
| **NSAID treatment**[a] | Prescription | M01A |

[a]Duration of prescriptions (days) was estimated as number of redeemed pills plus 25% for antiplatelet treatment and number of redeemed defined daily doses as stated in the Danish National Prescription Registry plus 25% for NSAID treatment. Days in which the patients were admitted to hospital were added to the duration.

© 2021 Fenger-Grøn M et al. *JAMA Network Open.*

eTable 4. Distribution of $CHA_2DS_2$-VASc scores in the study cohorts and the subgroups with bipolar disorder or schizophrenia.

| | **Study of treatment initiation (patients)** | | | **Study of treatment prevalence (entry years)** | | |
|---|---|---|---|---|---|---|
| | Bipolar disorder | Schizophrenia | Full cohort | Bipolar disorder | Schizophrenia | Full cohort |
| Values stated (unless otherwise specified) | N (%) | N (%) | N (%) | N (%) | N (%) | N (%) |
| Total, N | 1,208 | 572 | 147,810 | 7,954 | 3,259 | 1,002,721 |
| $CHA_2DS_2$VASc group | | | | | | |
| 2 | 254 (21.0) | 183 (32.0) | 32,599 (22.1) | 1,482 (18.6) | 895 (27.5) | 194,143 (19.4) |
| 3 | 322 (26.7) | 160 (28.0) | 39,063 (26.4) | 1,906 (24.0) | 777 (23.8) | 239,317 (23.9) |
| 4 | 291 (24.1) | 116 (20.3) | 36,829 (24.9) | 1,809 (22.7) | 699 (21.4) | 253,017 (25.2) |
| 5 | 184 (15.2) | 68 (11.9) | 21,094 (14.3) | 1,345 (16.9) | 503 (15.4) | 162,186 (16.2) |
| ≥6 | 157 (13.0) | 45 (7.9) | 18,225 (12.3) | 1,412 (17.8) | 385 (11.8) | 154,058 (15.4) |
| $CHA_2DS_2$VASc score, mean (sd) | 3.78 (1.43) | 3.39 (1.35) | 3.73 (1.41) | 4.01 (1.55) | 3.66 (1.47) | 3.92 (1.48) |

© 2021 Fenger-Grøn M et al. *JAMA Network Open.*

eTable 5: Characteristics of **matched references** for those with bipolar disorder or schizophrenia.

| N (%), and percentages among the exposed (from table 1) in square brackets unless otherwise stated | **Study of treatment initiation (references)** | | | | **Study of treatment prevalence (reference entry years)** | | | |
|---|---|---|---|---|---|---|---|---|
| | Bipolar disorder | | Schizophrenia | | Bipolar disorder | | Schizophrenia | |
| Total, N | 3,624[a] | | 1,716[b] | | 23,862 | | 9,777 | |
| Age in years, mean (sd) | 74.55 (10.26) | | 69.27 (12.32) | | 73.99 (10.36) | | 67.55 (12.68) | |
| Age group | | | | | | | | |
| <60 years | 290 (8.0) | [7.3] | 356 (20.7) | [19.9] | 5,611 (23.5) | [23.8] | 4,022 (41.1) | [41.0] |
| 60-69 years | 862 (23.8) | [25.1] | 487 (28.4) | [29.2] | 7,792 (32.7) | [32.7] | 2,949 (30.2) | [29.3] |
| 70-79 years | 1,318 (36.4) | [36.4] | 537 (31.3) | [31.6] | 6,975 (29.2) | [28.9] | 2,067 (21.1) | [22.2] |
| 80-89 years | 963 (26.6) | [25.7] | 282 (16.4) | [16.1] | 3,230 (13.5) | [13.5] | 684 (7.0) | [7.0] |
| ≥90 years | 191 (5.3) | [5.5] | 54 (3.1) | [3.1] | 254 (1.1) | [1.1] | 55 (0.6) | [0.5] |
| Sex | | | | | | | | |
| Female | 2,355 (65.0) | [65.0] | 1,065 (62.1) | [62.1] | 14,187 (59.5) | [59.5] | 5,496 (56.2) | [56.2] |
| Male | 1,269 (35.0) | [35.0] | 651 (37.9) | [37.9] | 9,675 (40.5) | [40.5] | 4,281 (43.8) | [43.8] |
| Period | | | | | | | | |
| 2005-2008 | 908 (25.1) | [25.3] | 429 (25.0) | [23.4] | 5,937 (24.9) | [24.9] | 2,106 (21.5) | [21.5] |
| 2009-2012 | 1,298 (35.8) | [35.0] | 589 (34.3) | [35.7] | 8,046 (33.7) | [33.7] | 3,144 (32.2) | [32.2] |
| 2013-2016 | 1,418 (39.1) | [39.7] | 698 (40.7) | [40.9] | 9,879 (41.4) | [41.4] | 4,527 (46.3) | [46.3] |
| Income[c] | | | | | | | | |
| 1st quartile | 1,458 (40.2) | [37.1] | 553 (32.2) | [43.9] | 9,030 (37.8) | [38.0] | 3,104 (31.7) | [41.1] |
| 2nd quartile | 1,168 (32.2) | [38.6] | 557 (32.5) | [45.6] | 7,777 (32.6) | [36.0] | 3,109 (31.8) | [47.3] |
| 3rd quartile | 554 (15.3) | [14.2] | 313 (18.2) | [7.5] | 3,625 (15.2) | [15.1] | 1,788 (18.3) | [8.2] |
| 4th quartile | 444 (12.3) | [10.1] | 293 (17.1) | [3.0] | 3,430 (14.4) | [11.0] | 1,776 (18.2) | [3.3] |
| Education level | | | | | | | | |
| ≤ 10 years | 1,968 (54.3) | [50.6] | 814 (47.4) | [64.2] | 12,224 (51.2) | [50.2] | 4,349 (44.5) | [59.6] |
| > 10 & ≤ 15 years | 1,214 (33.5) | [32.0] | 634 (36.9) | [27.4] | 8,455 (35.4) | [32.2] | 4,046 (41.4) | [30.3] |
| > 15 years | 442 (12.2) | [17.4] | 268 (15.6) | [8.4] | 3,183 (13.3) | [17.6] | 1,382 (14.1) | [10.1] |
| Migration history | | | | | | | | |
| Danish-born | 3,544 (97.8) | [97.8] | 1,667 (97.1) | [97.6] | 23,355 (97.9) | [97.9] | 9,488 (97.0) | [96.2] |
| Western immigrant | 44 (1.2) | [1.7] | 27 (1.6) | [0.9] | 195 (0.8) | [1.4] | 112 (1.1) | [2.0] |
| Non-western immigrant | 36 (1.0) | [0.5] | 22 (1.3) | [1.6] | 312 (1.3) | [0.7] | 177 (1.8) | [1.8] |
| Marital status | | | | | | | | |
| Unmarried | 238 (6.6) | [10.4] | 171 (10.0) | [37.9] | 1,387 (5.8) | [10.0] | 1,077 (11.0) | [36.1] |
| Married | 1,748 (48.2) | [35.0] | 885 (51.6) | [16.4] | 12,183 (51.1) | [38.0] | 5,297 (54.2) | [16.4] |
| Divorced | 491 (13.5) | [25.0] | 258 (15.0) | [30.6] | 3,132 (13.1) | [22.7] | 1,453 (14.9) | [33.4] |
| Widowed | 1,147 (31.7) | [29.6] | 402 (23.4) | [15.0] | 7,160 (30.0) | [29.3] | 1,950 (19.9) | [14.2] |
| *Comorbidity* | | | | | | | | |
| Congestive heart failure | 508 (14.0) | [17.1] | 213 (12.4) | [20.3] | 5,596 (23.5) | [26.9] | 2,370 (24.2) | [31.1] |
| Hypertension | 2,644 (73.0) | [67.8] | 1,232 (71.8) | [59.8] | 20,381 (85.4) | [82.2] | 8,425 (86.2) | [78.8] |
| Diabetes mellitus | 761 (21.0) | [24.5] | 396 (23.1) | [31.3] | 5,717 (24.0) | [27.2] | 2,555 (26.1) | [33.7] |
| Stroke/thromboembolism | 830 (22.9) | [24.8] | 388 (22.6) | [20.8] | 5,706 (23.9) | [24.7] | 2,514 (25.7) | [24.9] |
| Vascular disease | 884 (24.4) | [20.8] | 389 (22.7) | [21.5] | 6,323 (26.5) | [22.7] | 2,617 (26.8) | [21.8] |
| Kidney disease | 218 (6.0) | [11.4] | 118 (6.9) | [8.9] | 1,459 (6.1) | [10.9] | 720 (7.4) | [9.8] |
| Prior bleeding | 716 (19.8) | [25.5] | 294 (17.1) | [24.5] | 5,684 (23.8) | [28.4] | 2,011 (20.6) | [27.1] |
| Liver disease | 78 (2.2) | [3.9] | 37 (2.2) | [4.0] | 516 (2.2) | [3.8] | 247 (2.5) | [5.0] |
| Alcohol abuse | 164 (4.5) | [20.0] | 103 (6.0) | [25.5] | 1,074 (4.5) | [20.3] | 602 (6.2) | [26.3] |
| Other substance abuse | 19 (0.5) | [7.9] | 12 (0.7) | [11.0] | 75 (0.3) | [8.0] | 71 (0.7) | [11.6] |
| Dementia | 181 (5.0) | [15.5] | 63 (3.7) | [10.3] | 1,375 (5.8) | [18.0] | 322 (3.3) | [12.9] |
| Schizophrenia | 17 (0.5) | [8.1] | | | 85 (0.4) | [7.1] | | |
| Bipolar disorder | | | 9 (0.5) | [17.1] | | | 87 (0.9) | [17.4] |
| NSAID[d] treatment | 395 (10.9) | [10.1] | 166 (9.7) | [10.8] | 1,688 (7.1) | [7.6] | 698 (7.1) | [9.8] |
| Antiplatelet treatment | 1,701 (46.9) | [44.0] | 770 (44.9) | [42.0] | 9,415 (39.5) | [42.0] | 3,598 (36.8) | [42.7] |
| | | | Time since AF, Mean (SD) | | 6.77 (5.32) | [5.43] | 6.34 (5.22) | [4.89] |
| | | | 1 years | | 3,036 (12.7) | [12.7] | 1,422 (14.5) | [14.5] |
| | | | 2 years | | 2,622 (11.0) | [11.0] | 1,230 (12.6) | [12.6] |
| | | | 3 years | | 2,277 (9.5) | [9.5] | 1,089 (11.1) | [11.1] |
| | | | 4 years | | 2,067 (8.7) | [8.7] | 906 (9.3) | [9.3] |
| | | | ≥ 5 years | | 13,860 (58.1) | [58.1] | 5,130 (52.5) | [52.5] |

[a]Number of unique references in the sample was 3,568.
[b]Number of unique references in the sample was 1,701.
[c]Year-specific quartiles of the gross Danish population.
[d]Non-steroidal anti-inflammatory drugs

© 2021 Fenger-Grøn M et al. *JAMA Network Open.*
